# Use of Flattening Filter-Free Photon Beams in Treating Medulloblastoma: A Dosimetric Evaluation

**DOI:** 10.1155/2014/769698

**Published:** 2014-01-21

**Authors:** Pichandi Anchineyan, Ganesh K. Mani, Jerrin Amalraj, Balaji Karthik, Surega Anbumani

**Affiliations:** ^1^CyberKnife Center, HealthCare Global Enterprises, No. 7, HCG Tower 2, Kalinga Rao Road, Sampangiram Nagar, Bangalore 560 027, India; ^2^Department of Radiation Physics, Kidwai Memorial Institute of Oncology, Bangalore 560029, India

## Abstract

*Aim*. To evaluate the dosimetric benefits of flattening filter-free (FFF) photon beams in intensity modulated radiation therapy (IMRT) and Rapid Arc (RA) over conventional CSI methods. *Methods and Materials*. Five patients treated with IMRT using static multileaf collimators (MLC) were randomly selected for this retrospective study. Dynamic MLC IMRT, RA, and conformal therapy (3DCRT) were iterated with the same CT data sets with and without flattening filter photons. Total dose prescribed was 28.80 Gy in 16 fractions. Dosimetric parameters such as *D*_max⁡_, *D*_min⁡_, *D*_mean_, *V*_95%_, *V*_107%_, DHI, and CI for PTV and *D*_max⁡_, *D*_mean_, *V*_80%_, *V*_50%_, *V*_30%_, and *V*_10%_ for OARs were extracted from DVHs. Beam on time (BOT) for various plans was also compared. *Results*. FFF RA therapy (6F_RA) resulted in highly homogeneous and conformal doses throughout the craniospinal axis. 3DCRT resulted in the highest *V*_107%_ (SD) 46.97 ± 28.6, whereas flattening filter (FF) and FFF dynamic IMRT had a minimum *V*_107%_. 6F_RA and 6F_DMLC resulted in lesser doses to thyroid, eyes, esophagus, liver, lungs, and kidneys. *Conclusion*. FFF IMRT and FFF RA for CSI have definite dosimetric advantages over 3DCRT technique in terms of target coverage and OAR sparing. Use of FFF in IMRT resulted in 50% reduction in BOT, thereby increasing the treatment efficiency.

## 1. Introduction

Medulloblastoma is a fast growing tumor of the cerebellum (posterior fossa) that controls stability, posture, and complex motor functions such as verbal communication and swallowing.

About 400 new patients, primarily children, were diagnosed in the US every year, slightly more often in males than in females [1]. It is the most common brain tumor in children aged four and younger and the second most common brain tumor in children aged 5–14 years [2]. Subsequent to surgery, medulloblastoma is usually treated with CSI. Although radiation therapy had proven successful, investigators are still looking for new ways to mitigate the potential side effects of this treatment [2]. Treatment related late complications are usually hearing disability, declined cognition, cardiomyopathy, cataract formation, retarded growth, endocrine dysfunction, and second malignancies. Clinicians consider using techniques such as IMRT and RA that aim to converge beams of radiation directly at the tumor eventually improving the long term complications free survival. However, radiotherapy (RT) planning, delivery, and junction dose verification remain exigent for craniospinal irradiation (CSI) in medulloblastoma patients. Hence investigating the emerging new RT techniques such as FFF in IMRT and RA on the basis of dose volume parameters was encouraged to reduce the normal tissue complications [3].

Conventional two-dimensional planning for CSI involved field shaping using bony landmarks in X-ray radiographs; later it evolved into CT simulation techniques [4, 5]. Geometrical field matching was generally followed in such techniques without computing any dose volume data for the tumor and normal tissues. Modified treatment planning methods were adapted to get better tumor coverage, dose homogeneity, and conformity. The practicability of conventional linear accelerator (LA) IMRT for CSI in small children had been reported by Parker et al. [6]. The matching of cranial and spinal fields still poses a problem in adult patients with larger spinal lengths since it usually exceeds allowable maximum field size. Helical tomotherapy allows treatment to large cylindrical volumes (40 × 160 cm^2^) that was compromised with the longer BOT. It raises concerns about intrafraction motion and whole-body integral doses. When the FF was removed from the linear accelerators head, a marked increase in dose rate up to 1400 MU/min for 6 MV and 2400 MU/min for 10 MV beams is possible. The higher dose rate could make treatment delivery more accurate, by giving the patient less time to move between setup and treatment completion. This might be particularly helpful in CSI, where the tissues are far more mobile than in the cranium.

There is no dosimetric comparison between flattened and unflattened photon beams for CSI. The aim of this study is to determine the feasibility of using FFF beams in IMRT and RA for CSI in medulloblastoma patients and to dosimetrically compare it with 3DCRT, IMRT with static segments (6X_SMLC), IMRT with dynamic segments (6X_DMLC), Rapid Arc therapy (6X_RA) with FFF IMRT (6F_DMLC), and Rapid Arc therapy (6F_RA).

## 2. Methods and Materials

Patients were CT scanned from the vertex to coccyx in prone position using immobilization device (Orfit Industries n.v., Belgium) on multislice CT scanner (GE Healthcare, USA). Axial images of 3 mm slice thickness were exported to Mimvista contouring station (MIM software Inc, USA) where the target volumes (PTV_Brain, PTV_Spine) and normal structures were delineated by radiation oncologists as per the recommended guidelines [7]. PTV_Spine included the entire spinal canal, including cerebrospinal extension to spinal ganglia. OARs such as eyes, thyroid, heart, lungs, esophagus, liver, and kidney were outlined in the axial CT sections. Treatment planning was performed in Eclipse (Version 11.0; Varian Associates, Palo Alto, CA, USA) treatment planning system (TPS). It is configured for both true beam millennium 120 multileaf collimator (MLC) and Siemens ARTISTE 160 MLC treatment units. The range of patients' spine length varied from 28.52 cm to 43.75 cm (median length: 33.4 cm). A maximum field size of 40 × 40 cm^2^ can be possible with the 120 millennium MLC and 160 MLC Artiste. Anisotropic analytical algorithm was the dose calculation algorithm used for inverse optimization. We used the CT data set of five randomly selected medulloblastoma patients (median age: 10 yrs), previously treated with conventional IMRT for this retrospective study. Conventional 3DCRT plan, 6X_DMLC, 6F_DMLC, 6X_RA, and 6F_RA were iterated which resulted in six plans for each patient. The total dose prescribed was 28.80 Gy in 16 fractions with 1.8 Gy per fraction. An evaluation criterion of 98% of the PTV receiving 100% of the prescription dose and 107% maximum dose was followed as per our institution protocol. Normal tissue sparing was considered as important as the tumor coverage.

### 2.1. Radiotherapy with Conformal Photon Beams (3DCRT)

**Figure 1:**
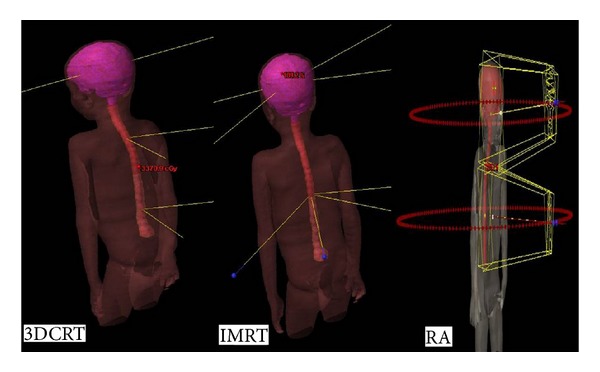
Beam arrangements for 3DCRT, IMRT, and RA.

The 3DCRT for CSI comprised three separate treatment plans such as 3d_Brain, 3d_Spine1, and 3d_Spine2. For the whole brain irradiation, 6 MV photon beam was collimated in such a way that the spine field's divergence can be easily matched. Spine 1 comprised the region between 2nd cervical vertebra, 10th thoracic vertebra and whereas spine 2 was between 11th thoracic vertebra and 5th lumbar vertebra. Spinal cord treatments were planned with two oblique beam portals 330° and 30°. The 25° enhanced dynamic wedges were used to avoid high-dose regions falling beneath the skin and to improve dose coverage at larger depths. For the three plans, depth from skin where the maximum possible coverage achieved was taken as the reference point for dose normalization. Plans were summed up in evaluation mode of the TPS to analyze the junction dose. The sagittal view of the 3DCRT beam arrangement is shown in Figure 1.

### 2.2. Intensity Modulated Radiation Therapy (IMRT) Planning

IMRT confines the radiation dose more precisely to target alone. This is achieved by modulating or controlling the radiation beam intensity in multiple beamlets. It also allows higher radiation doses to be focused on regions within the tumor while minimizing the dose to surrounding OARs. IMRT delivery methods using conventional MLCs can be realized in several ways: (1) “step-and-shoot” static IMRT using multiple MLC shapes and (2) dynamic IMRT with fixed gantry and moving MLC leaves. For CSI, jagged junction or intensity feathering technique was used to plan IMRT and RA plans. In this technique, 6 MV photon beams with same optimization can be iterated (PTV) with multiple isocenters. Thus, summing up of two or three plans was not needed. The junction evaluation can be avoided which could be a tedious process involving suitable collimator angles to match dose gradients from the adjacent field. Since there was no beam matching involved, this treatment technique is less likely to produce hot or cold spots at the junction, compared to conventional techniques. Except for one tallest patient, all other cases were planned with two isocenters and 8 gantry angles. For the tallest of the patients, entire spine was split into three regions and two separate isocenters apart from the cranial junction were planned with 12 beam portals. Figure 1 shows IMRT beam arrangement. Beam geometry consisted of four coplanar fields for the whole skull with the gantry angles 225°, 115°, 310°, and 50° and upper spine with gantry angles 20°, 50°, 340°, and 310°. In case of an additional isocenter for the tallest of all patients, lower spine gantry angles are 0°, 30°, and 60°. Default smoothing values were used during optimization. To improve the results, efforts were made to modify constraints and priority factors in IMRT plans.

### 2.3. Rapid Arc Therapy (RA)

RA optimization was performed with version 11.0 from Eclipse (Varian, Palo Alto, CA, USA). The maximum dose rate (DR) of 600 MU/min for 6X_RA and DR of 1600 MU/Min for 6F_RapidArc was selected. All plans were done with 2 isocenters and 2 full Arcs (179°–181°) for each isocenter (Figure 1). These two Arcs were delivered in opposite rotations (clockwise and counterclockwise). Collimator was set to rotate to a value other than zero in order to avoid tongue and groove effect. The anisotropic analytical algorithm (AAA, version 11.00) was the dose calculation algorithm used.

### 2.4. Dose-Volume Analysis

Target coverage was quantified with the conformity index (CI) based on International Commission of Radiation Units report: 50 (ICRU 50). The dose homogeneity index (DHI) was calculated using the formula coined by Wu et al. [8]. The dosimetric parameters such as *D*_max⁡_, *D*_mean_, *V*_2%_, *V*_98%_, *V*_95%_, and *V*_107%_ were evaluated for the six planning techniques. The volumes of each OAR receiving >80% (high; *V*_80%_), >50% (intermediate; *V*_50%_), >30% (low; *V*_30%_), and >10% (low; *V*_10%_) of the prescribed dose were extracted from the DVH and compared among the techniques. The techniques were evaluated for average total BOT.

## 3. Results

**Figure 2:**
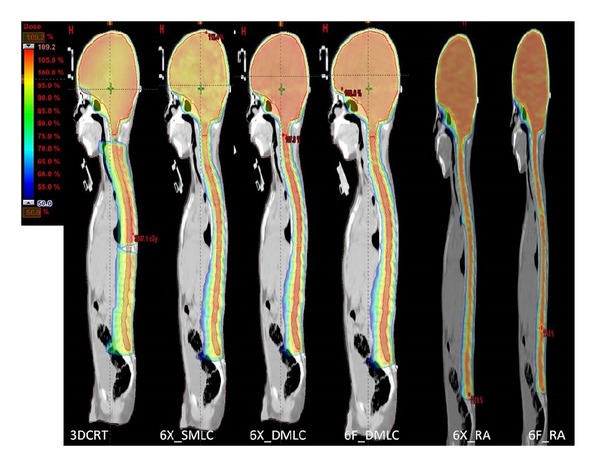
Dose distribution from 3DCRT, IMRT, and RA techniques, sagittal view.

The sagittal dose distribution resulted from 3DCRT, IMRT, and RA techniques was shown in Figure 2. Among the six techniques, 3DCRT resulted in maximum dose heterogeneity. 6X_DMLC and 6F_RA lead to more homogeneous and conformal doses throughout the craniospinal axis. Plan dosimetric parameters related to target coverage, homogeneity, and conformity resulting from the six techniques were presented in Table 1.

*D*_max⁡_, *D*_mean_, *V*_2%_, *V*_98%_, and *V*_95%_ values obtained in each method were almost similar. 3DCRT had lesser minimum dose to target *D*_min⁡_ compared to other methods. It resulted in the highest *V*_107%_ (SD) 46.97 ± 28.6, whereas FF and FFF dynamic IMRT had a minimum *V*_107%_. Dose statistics for maximum mean dose (*D*_mean_) for OARs were listed (Table 2).

**Figure 3:**
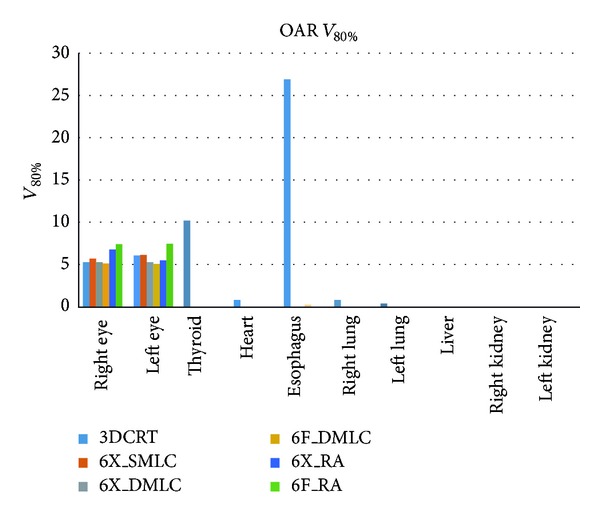
Graphical plot: *V*_80%_ for OARs.

**Figure 4:**
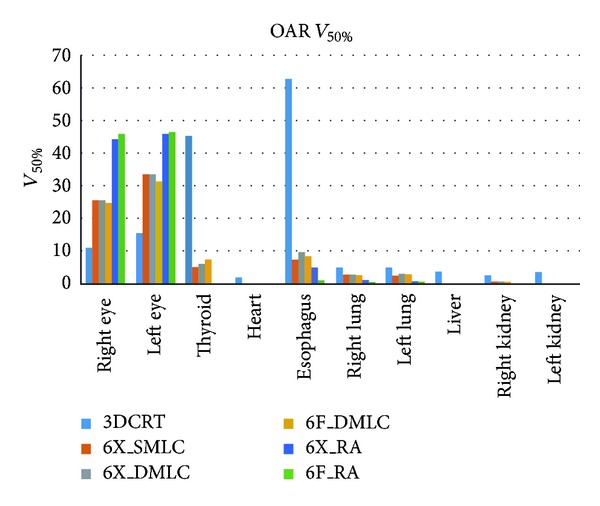
Graphical plot: *V*_50%_ for OARs.

There was no significant difference between OAR doses resulted from 6X_DMLC, 6F_DMLC, 6X_RA, and 6F_RA plan except mean dose to lungs and eyes. The mean lungs dose from 6X_SMLC was lesser (4.78 ± 0.73 Gy) than 6F_RA (5.93 ± 0.72 Gy) and 6X_RA (6.01 ± 72 Gy). The mean dose to eyes was 14.88 Gy (6F_RA) and 7.87 Gy (3DCRT). The percentage volumes of each OAR receiving *V*_80%_ and *V*_50%_ of radiation from the three different treatment planning techniques were presented in Figures 3 and 4, respectively.

IMRT (FF/FFF) and RA (FF/FFF) reduction reduces the amount of OAR volume receiving doses such as 80%, 50%, and 30%. Mean dose, *V*_80%_, *V*_50%_, and *V*_30%_ for thyroid, heart, esophagus, lungs, liver, and kidneys were similar in all the techniques except 3DCRT. Very low thyroid doses were achievable with RA therapy (4.71 Gy (6X_RA) and 5.10 Gy (6F_RA)). Lower values of *V*_10%_ for OARs were possible with IMRT compared to RA technique. Average BOT was 3.43 min (6X_DMLC), 1.59 min (6F_DMLC), 5 min (6X_RA), and 4.5 min (6F_RA) compared to 3DCRT (1.262 min).

## 4. Discussions

Dosimetric parameters for PTV were almost similar in all techniques except the minimal target dose (*D*_min⁡_). A minimum dose of 5.76 Gy (3DCRT) was the least compared to others. Thus, CSI with 3DCRT could lead to lesser target coverage. IMRT (FF/FFF) and RA therapy (FF/FFF) have led to eye doses that were within the tolerance limit (RTOG 0225).

Hypothyroidism is the most common complication observed after RT. Thyroid gland is viewed as a radiation-resistant organ though the range of thyroid-ablative radiation doses seems to be wide, being 10–80 Gy according to Foo et al. [9]. Theoretically the development of hypothyroidism in RT patients would primarily depend on *V*_30%_, the volume receiving relatively high radiation doses (≥30 Gy) thus with the risk of insufficient post-RT hormone production. This volume might show considerable interpatient variation, as the size of the thyroid gland might vary from patient to patient. However, to our knowledge, no study had evaluated the association between the thyroid volume exposed to high-dose irradiation and the development of post-RT hypothyroidism in CSI planned with FFF beams. The use of FFF in IMRT and RA for CSI could reduce the risks of hypothyroidism. Also, the late risks such as cardiomyopathy, liver diseases, renal failure, and esophagitis could be eliminated using FFF beams, due to considerable reduction in doses deposited in OARs.

Acceptable dose to eyes and lesser doses to other critical organs were possible with FFF IMRT and FFF RA therapy. 3DCRT leads to higher values of *D*_max⁡_ and *D*_mean_ that could cause late toxicity (4). *V*_80%_ was similar in all the techniques for eyes and other normal structures. The highest *V*_80%_ (26.97% in esophagus) resulted from 3DCRT. IMRT (FF/FFF) and RA (FF/FFF) techniques lead to zero percentage of *V*_80%_ which could be more clinically relevant in sparing the OARs. Lesser amount of normal tissues received 50% and 30% doses in IMRT (FF/FFF) and RA (FF/FFF) compared to 3DCRT.

Treatment delivery efficiency is quantified by lesser BOT. 6X_DMLC IMRT delivery required more treatment time. 3DCRT and 6X_SMLC showed no difference in BOT. FFF IMRT/FFF RA had lesser beam on time that improves the efficiency of therapy, by minimizing patient movement and intrafraction variation errors in treatment setup.

## 5. Conclusion

Using FFF beams in IMRT/RA therapy for CSI had definite dosimetric advantages in target coverage and OAR sparing over flattened photon beam therapy. Lesser BOT achievable with FFF beams improves efficiency of CSI radiotherapy. In addition, high precision techniques evade the concern over junction doses due to minimal set-up errors. Hence, the use of FFF beams is feasible and effective in treating medulloblastoma patients.

## Figures and Tables

**Table 1:** Dosimetric parameters for combined target volumes (brain and spine).

Combined target volumes (brain and spine)												
Dosimetric parameters	3DCRT		6X_SMLC		6X_DMLC		6F_DMLC		6X_RA		6F_RA	
	Mean	SD	Mean	SD	Mean	SD	Mean	SD	Mean	SD	Mean	SD
*D*_max⁡_	34.31	0.81	32.91	0.67	31.97	0.91	31.87	0.41	32.26	0.61	32.62	0.63
*D*_min⁡_	5.76	1.45	20.49	1.82	19.77	2.63	19.35	3.24	21.54	2.61	22.61	1.80
*D*_mean_	30.78	0.68	30.09	0.14	30.11	0.11	30.15	0.11	30.77	0.43	30.82	0.80
*D*_2%_	32.38	0.68	31.34	0.23	30.75	0.10	30.78	0.18	31.42	0.52	31.68	0.83
*D*_98%_	28.51	0.46	28.80	0.00	28.80	0.00	28.80	0.00	28.79	0.02	28.53	0.57
*V*_95%_	99.12	0.27	99.62	0.13	99.76	0.09	99.77	0.12	99.83	0.10	99.55	0.62
*V*_107%_	46.97	28.60	10.94	4.90	1.23	0.70	2.88	3.74	49.15	30.84	66.36	21.59
DHI	13.45	2.33	8.81	0.80	6.78	0.36	6.89	0.62	9.12	1.78	10.92	1.74
CI	1.19	0.08	1.10	0.03	1.09	0.03	1.10	0.04	1.04	0.02	1.05	0.01

**Table 2:** Mean dose data for OARs.

OAR												
	*D*_mean_ in Gy											
Dosimetric parameters	3DCRT		6X_SMLC		6X_DMLC		6F_DMLC		6X_RA		6F_RA	
	Mean	SD	Mean	SD	Mean	SD	Mean	SD	Mean	SD	Mean	SD
Rt eye	10.95	11.68	10.31	3.32	10.58	3.31	9.99	3.56	14.64	0.84	14.88	2.39
Lt eye	7.87	3.93	11.65	2.57	11.73	2.61	11.35	2.79	14.43	1.07	14.91	2.64
Thyroid	15.28	3.9	7.36	3.16	8.68	2.53	8.56	2.99	4.71	0.44	5.10	1.07
Heart	7.24	2.15	2.81	0.77	3.08	0.77	4.09	3.01	3.62	0.58	3.56	0.66
Esophagus	17.16	5.88	8.34	2.01	8.06	1.94	8.62	1.91	7.63	1.13	7.25	1.27
Rt lung	4.98	0.73	4.78	0.73	4.85	0.84	4.63	0.85	6.01	0.72	5.93	0.72
Lt lung	4.87	1.37	4.76	1.69	5.05	1.91	4.81	1.81	5.89	0.74	5.90	0.90
Liver	5.21	1.2	3.34	0.37	3.47	0.4	3.36	0.35	4.42	0.35	4.46	0.27
Rt kidney	4.26	1.16	2.83	0.7	3.39	0.83	3.15	0.78	4.69	1.04	4.76	1.14
Lt kidney	4.43	1.57	2.39	0.31	2.96	0.42	2.66	0.37	4.84	0.86	4.78	0.92

## References

[1] Packer RJ, Gajjar A, Vezina G (2006). Phase III study of craniospinal radiation therapy followed by adjuvant chemotherapy for newly diagnosed average-risk medulloblastoma. *Journal of Clinical Oncology*.

[2] Gajjar A, Hernan R, Kocak M (2004). Clinical, histopathologic, and molecular markers of prognosis: toward a new disease risk stratification system for medulloblastoma. *Journal of Clinical Oncology*.

[3] Dolecek TA, Propp JM, Stroup NE, Kruchko C (2012). CBTRUS statistical report: primary brain and central nervous system tumors diagnosed in the United States in 2005–2009. *Neuro-Oncology*.

[4] Robinson GW (2012). *Medulloblastoma*.

[5] Sharma SD, Gupta T, Jalali R, Master Z, Phurailatpam RD, Sarin R (2009). High-precision radiotherapy for craniospinal irradiation: evaluation of three-dimensional conformal radiotherapy, intensity-modulated radiation therapy and helical Tomotherapy. *The British Journal of Radiology*.

[6] Parker W, Filion E, Roberge D, Freeman CR (2007). Intensity-modulated radiotherapy for craniospinal irradiation: target volume considerations, dose constraints, and competing risks. *International Journal of Radiation Oncology Biology Physics*.

[7] International Commission on Radiation Units and Measurements (ICRU) (1999). Prescribing, recording and reporting photon beam therapy. *ICRU Report*.

[8] Wu QJ, Yoo S, Kirkpatrick JP, Thongphiew D, Yin FF (2009). Volumetric Arc intensity-modulated therapy for spine body radiotherapy: comparison with static intensity-modulated treatment. *International Journal of Radiation Oncology Biology Physics*.

[9] Foo ML, McCullough EC, Foote RL, Pisansky TM, Shaw EG (1993). Doses to radiation sensitive organs and structures located outside the radiotherapeutic target volume for four treatment situations. *International Journal of Radiation Oncology Biology Physics*.

